# Cholesterol Balance in Prion Diseases and Alzheimer’s Disease

**DOI:** 10.3390/v6114505

**Published:** 2014-11-20

**Authors:** Samia Hannaoui, Su Yeon Shim, Yo Ching Cheng, Erica Corda, Sabine Gilch

**Affiliations:** Department of Ecosystem and Public Health, Faculty of Veterinary Medicine, and Hotchkiss Brain Institute, University of Calgary, 3280 Hospital Drive Northwest, Calgary, AB T2N 4Z6, Canada; E-Mails: shannaou@ucalgary.ca (S.H.); syshim@ucalgary.ca (S.Y.S.); ycchen@ucalgary.ca (Y.C.C.); ecorda@ucalgary.ca (E.C.)

**Keywords:** prions, prion disease, Alzheimer’s disease, neurodegenerative diseases, cholesterol, cholesterol imbalance, lipid rafts, therapy

## Abstract

Prion diseases are transmissible and fatal neurodegenerative disorders of humans and animals. They are characterized by the accumulation of PrP^Sc^, an aberrantly folded isoform of the cellular prion protein PrP^C^, in the brains of affected individuals. PrP^C^ is a cell surface glycoprotein attached to the outer leaflet of the plasma membrane by a glycosyl-phosphatidyl-inositol (GPI) anchor. Specifically, it is associated with lipid rafts, membrane microdomains enriched in cholesterol and sphinoglipids. It has been established that inhibition of endogenous cholesterol synthesis disturbs lipid raft association of PrP^C^ and prevents PrP^Sc^ accumulation in neuronal cells. Additionally, prion conversion is reduced upon interference with cellular cholesterol uptake, endosomal export, or complexation at the plasma membrane. Altogether, these results demonstrate on the one hand the importance of cholesterol for prion propagation. On the other hand, growing evidence suggests that prion infection modulates neuronal cholesterol metabolism. Similar results were reported in Alzheimer’s disease (AD): whereas amyloid β peptide formation is influenced by cellular cholesterol, levels of cholesterol in the brains of affected individuals increase during the clinical course of the disease. In this review, we summarize commonalities of alterations in cholesterol homeostasis and discuss consequences for neuronal function and therapy of prion diseases and AD.

## 1. Introduction

Transmissible spongiform encephalopathies (TSEs) or prion diseases affect both humans and animals [1,2,3]. Human prion diseases are highly heterogeneous and include sporadic (sporadic Creutzfeldt-Jakob disease (CJD)), genetic (genetic CJD, Gerstmann-Sträussler-Scheinker syndrome, Fatal Familial Insomnia) and infectiously acquired forms (Kuru, variant CJD). Animal TSEs include scrapie in sheep and goat, chronic wasting disease in deer, elk and moose, and bovine spongiform encephalopathy in cattle.

Typically, TSEs are characterized by vacuolation (spongiosis) of the neuropil, gliosis, and neuronal loss. Furthermore, the pathogenesis of prion diseases is usually associated with an abnormal and progressive accumulation of PrP^Sc^, a misfolded isoform of the cellular prion protein PrP^C^ [4,5].

Two models were proposed to explain the mechanism of PrP^C^ conversion into PrP^Sc^. In the template-directed refolding model or autocatalytic model, PrP^C^ to PrP^Sc^ conversion needs a folding intermediate, designated PrP*. PrP^Sc^ monomers or dimers form a complex with PrP*, which may lower the activation energy barrier for the formation of new PrP^Sc^ [6]. In the now widely accepted non-catalytic nucleated polymerization model [7], PrP^C^ to PrP^Sc^ conversion is possible through the formation of PrP^Sc^ cores. In this model, PrP^C^ conversion into PrP^Sc^ is a reversible process, however, at equilibrium it strongly favors the PrP^C^ isoform. The first step of nucleation generates multimers of PrP^Sc^, is slow and reversible, and precedes a second step which allows the recruitment of PrP^C^ monomers by PrP^Sc^ polymers (core), followed by their conversion into PrP^Sc^ [8]. Once a seed is present, further monomer addition of PrP^C^ is accelerated and the process is irreversible.

Though PrP^C^ is essential for the development and the progression of prion disease [9], its function is still unknown. Nevertheless, evidence is provided that PrP^C^ is involved in neuro-protective and anti-apoptotic functions, and has a role in structure and maintenance of synaptic plasticity, cell survival, proliferation and neurite outgrowth [10,11,12,13]. In addition, recent data suggest that PrP^C^ is involved in amyloid β (Aβ) peptide neurotoxicity by acting as a receptor for Aβ peptide oligomers [14].

PrP^C^ is a ubiquitously expressed glycoprotein, with highest levels found in the central nervous system (CNS) [15,16,17,18]. It is linked to the plasma membrane by a glycosyl-phosphatidyl-inositol- (GPI-) anchor [19]. Like many other GPI-anchored proteins, both PrP^C^ and PrP^Sc^ are found associated with lipid rafts [20,21], detergent resistant membrane domains (DRMs) enriched in cholesterol and glycosphingolipids [22].

PrP^C^ undergoes internalization, however, the mechanism is still debated because several pathways, such as rafts/caveolae, caveolae-like and clathrin-dependent endocytosis were reported to be involved [21,23,24,25,26,27]. Once internalized, only 10% of PrP^C^ is degraded [28], whereas the majority is recycled to the plasma membrane [29,30]. The functional significance of the recycling process is still unknown, however, endocytosis and recycling processes appear to be essential for PrP^Sc^ formation [31,32,33].

The subcellular compartment of prion conversion is still not entirely elucidated. Several sites of conversion were proposed, including plasma membrane [34,35], endolysosomal vesicles [36,37] and endoplasmic reticulum (ER) [38,39,40]. More recently, two independent studies performed by the groups of Zurzolo and Peters demonstrated that recycling endosomes are a potential site of prion conversion [41,42]. However, a huge body of evidence suggests that cell surface localization of PrP^C^ is required for conversion into PrP^Sc^ [31,43,44,45,46], and that the first contact between PrP^C^ and PrP^Sc^ occurs at the plasma membrane [47].

It should be highlighted that both PrP^C^ and PrP^Sc^ are found in lipid rafts when isolated from either cell lines or mouse brain homogenate [21,43,48,49,50]. Directing PrP^C^ to non-lipid raft membrane domains by replacing the signal peptide mediating GPI-anchor attachment with transmembrane domains of other proteins prevents the formation of PrP^Sc^ [23,48]. Similarly, destabilization of lipid rafts by cholesterol depletion blocks PrP^C^ to PrP^Sc^ conversion [48,51,52]. Nevertheless, the role of lipid rafts in PrP^Sc^ formation remains unclear. These microdomains may be involved in gathering PrP^C^ and PrP^Sc^, contain a crucial co-factor essential for the conversion, or permit the co-internalization of PrP^C^ and PrP^Sc^, thereby promoting the conversion of PrP^C^ into PrP^Sc^ (for review [39]).

## 2. Cholesterol Metabolism

Cholesterol is a lipid molecule of the sterol family which plays an important role in the structure and function of membranes, in particular, the plasma membrane. Cholesterol plays a key role in cell growth and viability, and it represents 20%–25% of all lipids. A dysregulation of cerebral cholesterol homeostasis is a significant risk factor in several neurodegenerative diseases, e.g., Alzheimer’s, Parkinsons’s and Huntington’s disease (AD, PD and HD; [53]).

### 2.1. Synthesis and Uptake

**Figure 1:**
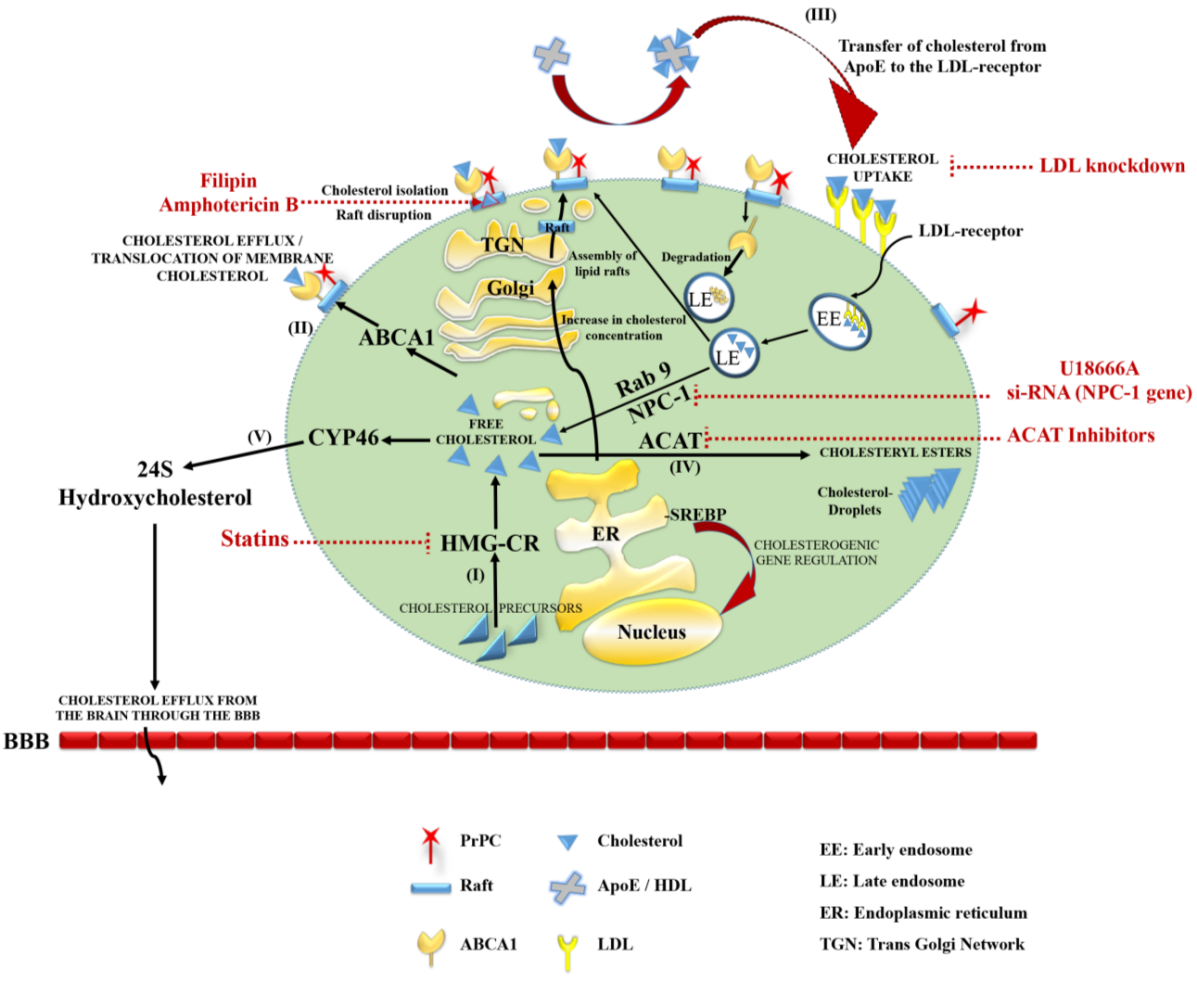
Cholesterol metabolism, lipid rafts and cellular prion propagation. (I) Cellular cholesterol, an essential component of lipid rafts, is synthesized from cholesterol precursors, with HMGCR as the rate limiting enzyme in the ER membrane; (II) Synthesized Cholesterol is secreted in an ABCA1 dependent process to form lipoprotein particles, or it is incorporated into membranes of the secretory pathway, where lipid rafts are assembled in the trans-Golgi network; (III) Uptake of extracellular cholesterol is mediated by ApoE/HDL interaction with the LDL-receptor. Cholesterol dissociates from the lipoprotein, is de-esterified and exported from late endosomes in a NPC protein dependent manner. It is then either transported to the plasma membrane or adds to the regulatory pool which prevents cleavage and nuclear translocation of SREBP. Once the capacity of cells to absorb cholesterol is exceeded, cholesterol is, in a small part, esterified by ACAT and accumulated as cytosolic lipid droplets (IV); The major pool of excess cholesterol is hydroxylated by CYP46 and converted to 24S-hydroxycholesterol, crosses the BBB and diffuses into the blood circulation (V). The red dotted lines represent examples for interference in cholesterol metabolism that resulted in a reduction or clearance of cellular PrP^Sc^ accumulation.

Intracellular cholesterol levels are well regulated by a feedback loop and depend on its synthesis, uptake, and transport, but also on its metabolism and catabolism (Figure 1). Cholesterol is synthetized from acetyl-Coenzyme A (Acetyl-CoA) via the mevalonate pathway (HMG-CoA reductase pathway) through several intermediates. The rate limiting step is the conversion of HMG-CoA to mevalonate by the enzyme HMG-CoA reductase (HMGCR) in the ER membrane. The ER is crucial for the regulation of cholesterol homeostasis. By using an indirect, ACAT activity-dependent assay, Lange and co-workers [54] measured how the ER pool of cholesterol responds to cholesterol loading in human fibroblasts. A decrease in cellular cholesterol allows SCAP/SREBP (SREBP Cleavage-Activating Enzyme/Sterol Regulatory Element-Binding Proteins) to leave the ER and to be transported to the Golgi apparatus. SREBP is then cleaved by two proteases, S1P and S2P (site-1 and -2 protease). The *N*-terminal domain of SREBP is translocated to the nucleus and acts as a transcription factor that activates several genes involved in cholesterol biosynthesis, in particular, HMGCR [55].

Cells can also take up cholesterol from the extracellular environment. Lipid particles are trapped by LDL receptors (LDL-R) which recognize apolipoproteins. After endocytosis of lipoproteins and hydrolysis of cholesterol esters, free cholesterol is integrated in the metabolically active pool of the cell. This process requires the presence of Niemann Pick C1 and C2 proteins, which mediate cholesterol transfer out of the endosomal system. Impairment of either of these proteins leads to an accumulation of cholesterol esters in late endosomes [56], thereby causing a syndrome known as Niemann Pick type C disease which is associated with neurodegeneration and dementia.

### 2.2. Storage and Elimination

Accumulation of free cholesterol is toxic for cells [57] and to avoid this situation, cholesterol is esterified by Acyl-Coenzyme A: cholesterol acyl-transferase 1 (ACAT1) and stored in the form of cytosolic lipid droplets. In contrast, the release of cholesterol from lipid droplets is catalyzed by cholesterol ester hydrolase (CEH). Efflux of cellular cholesterol is enabled by two mechanisms. The first is by passive diffusion, in which cholesterol is desorbed down the concentration gradient onto acceptor molecules. The second is an apolipoprotein-dependent pathway involving the membrane protein ABCA-1.

### 2.3. Cellular Cholesterol Localization and Trafficking

Cholesterol is enriched at the plasma membrane, particularly in lipid rafts. It is also abundant in endosomes and Golgi apparatus, especially in the trans-Golgi network where lipid rafts are assembled [58]. In contrast, the ER harbors only around 1% of cellular cholesterol. Cholesterol affects several cellular processes by interacting with other membrane lipids and specific proteins. High membrane cholesterol content decreases membrane fluidity and permeability [59,60,61,62].

### 2.4. Cholesterol in the Brain

In contrast to other organs, the brain is isolated from plasma derived cholesterol since lipoproteins are poorly or not at all capable to cross the intact blood-brain barrier (BBB). Therefore, nearly all cholesterol in the brain is synthesized *in situ* [63].

#### 2.4.1. Synthesis: Neurons and Astrocytes Interact in Cholesterol Homeostasis

The primary source of cerebral cholesterol is *de novo* synthesis by glial cells. Neurons are able to synthesize cholesterol only during the embryonic development. Later, glial lipoproteins become the main source of neuronal cholesterol in the CNS. There are two major pools where cholesterol is present: cholesterol in myelin produced by oligodendrocytes (glial cells) and cholesterol in the plasma membrane of neurons and astrocytes. Neuronal cholesterol is mainly synthesized by astrocytes [64]. External cholesterol is used by neurons for growth [65] and the development of a synaptic network [66], and also depends on neuronal types.

Astrocytes also synthetize and secrete HDL, the most abundant lipoproteins in the CNS, and Apolipoprotein E (ApoE), that enable cholesterol transport within the CNS [67]. Cholesterol is associated with ApoE by ABCA1, and this complex is excreted and found within HDL particles. Neurons capture these lipoproteins via LDL-R, SR-BI or other receptors of lipoproteins.

#### 2.4.2. Cholesterol Elimination from the Brain

Cholesterol cannot pass directly through the BBB, therefore, it is first converted to 24S-hydroxycholesterol (24S-OH) by cholesterol 24-hydroxylase (CYP46A1) [68,69,70,71]. Under normal conditions, this enzyme is only present in neuronal cells [72]. 24S-OH is excreted by neurons via ABCG1 and ABCG4 to ApoE molecules or to the cerebrospinal fluid [73] and can be recaptured by astrocytes where it can regulate cholesterol and ApoE synthesis [73,74]. 24S-OH in excess, which cannot be recaptured, crosses the BBB to be eliminated via the plasma [75].

## 3. Role of Cholesterol in the Formation of Microdomains

Cholesterol has the key role in the lateral organization of lipid membranes, in the form of specialized microdomains known as rafts [22,76,77,78]. In the outer leaflet of the membrane, the long and saturated acyl chains of sphingolipids strongly intercalate with cholesterol resulting in the dense-organization of lipid ordered phases in the membrane [79]. Cholesterol- and sphingolipid-rich microdomains are bordered by a lightly packed lipid disordered phase of unsaturated phospholipids. Distinct proteins can selectively partition into lipid rafts, thus it is thought that lipid rafts serve as specific protein sorting sites [77]. Lipid rafts are assumed to be involved in intracellular trafficking of proteins and lipids, secretory and endocytotic pathways as well as signal transduction pathways [60,80]. Rafts are detergent-resistant membrane domains which can be isolated when membranes are treated with mild detergents [81]. The first evidence of their existence in membranes came from studying model bilayer membranes. Indeed, observation by confocal fluorescence microscopy using lipophilic dyes showed separation of lipid disordered and ordered sphingomyelin-enriched phases in giant unilamellar vesicles prepared from phosphatidylcholine, cholesterol and sphingomyelin [82,83]. Two-photon fluorescent microscopy probing for lipid rafts in living cells detected a cholesterol-dependent increase in the appearance of fat-like domains, thereby providing support for the lipid raft hypothesis [84].

## 4. Cholesterol and Prions

Cholesterol is structurally important for lipid raft formation, and both PrP^C^ and PrP^Sc^ are associated with these membrane domains. Consequently, many studies in prion research focus on the relationship between cholesterol metabolism and PrP^Sc^/prion propagation.

The first evidence that cholesterol may have an impact on prion diseases was provided in 1965. Mould and co-workers pointed out that even if total cholesterol levels in cerebrospinal fluid remained relatively constant, a small rise occurred in goats clinically affected with scrapie [85]. Since then, numerous studies have been performed to investigate the effect of cholesterol on PrP^C^ processing, PrP^Sc^ formation and prion propagation (Figure 1).

### 4.1. Cholesterol-Enriched Lipid Rafts and Prion Protein Isoforms

Cell-free systems highlight the role of lipid rafts in prion conversion. Indeed, in a cell-free conversion assay, PrP^Sc^ formation was inhibited when PrP^C^ was associated with lipid rafts. This could be reversed by enzymatic release of PrP^C^ from lipid rafts, and also when exogenous PrP^res^ was inserted into contiguous membrane using polyethylene glycol for fusion [86]. Surprisingly, PrP^sen^ lacking the GPI-anchor (GPI^−^), was efficiently converted to PrP^res^ without any treatment. Using another cell-free system, the protein misfolding cyclic amplification (PMCA), it has been demonstrated that membrane binding is not necessary for an efficient conversion of PrP^C^ into PrP^Sc^ [87]. Nevertheless, integration of PrP^C^ into the plasma membrane appears to be critical for PrP^Sc^ toxicity, since transgenic mice overexpressing anchorless PrP do not develop clinical prion disease despite high levels of PrP^Sc^ present in the CNS [88]. Furthermore, studies involving postnatal neuronal knockout of PrP^C^ in infected mice demonstrated that PrP^Sc^ accumulates at high levels, however, it is not toxic to neurons in the absence of neuronal PrP^C^ [89]. Marella and co-workers reported that shedding of PrP^C^ induced by filipin treatment in infected N2a cells inhibited the conversion of PrP^C^ into PrP^Sc^ [24]. These reports indicated the dissociation between PrP^Sc^ formation and disease progression [88,90]. Therefore, for development of clinical disease, PrP^Sc^ has to be close to neuronal PrP^C^ in a contiguous compartment. Here, it is likely that lipid raft localization of PrP isoforms in particular plays a key role. Furthermore, PrP^C^ was shown to be related to signaling platforms, including several intracellular effectors, such as Fyn, Lyn, PI3K/Akt, PKA, ERK1/2, GSK3β and TACE among others [91,92,93,94,95]. These effectors and the signaling pathways in which they are involved may be affected when PrP^C^ is converted into PrP^Sc^. The disruption of PrP^C^-signaling events may lead to the loss of its neuroprotective function and/or to the gain of a neurotoxic function.

### 4.2. How does Interference in Cholesterol Metabolism Influence PrP^Sc^ Propagation?

#### 4.2.1. Inhibition of Cholesterol Synthesis

In 1995, a seminal study performed by A. Taraboulos and colleagues demonstrated that depletion of cholesterol from prion-infected N2a cells interferes with PrP^C^ processing and PrP^Sc^ propagation [48]. When cholesterol synthesis was inhibited by the treatment with lovastatin (also called mevinolin), a competitive inhibitor of HMGCR, PrP^Sc^ formation was decreased [48]. This could be due to the unavailability of a well folded mature PrP^C^, the indispensable substrate for the formation of PrP^Sc^. Indeed, it was shown that cholesterol depletion by mevinolin is responsible for misfolding of immature PrP, the main precursor of mature PrP [96,97]. Here, it was demonstrated that mature and immature diglycosylated isoforms of PrP^C^ are localized in different lipid compartments, and are distinctly sensitive to cholesterol and sphingolipids. When PrP^C^ overexpressing epithelial Fischer Rat Thyroid (FRT) cells were depleted of cholesterol using a combination of mevinolin and βCD (beta-cyclodextrine), or of sphingolipid, using fumonisin B1 (FB1), flotation of mature PrP^C^ was reduced in both conditions. Interestingly, the immature PrP^C^ isoforms behave differently. Cholesterol depletion leads to an increased flotation while sphingolipid depletion has the opposite effect, it drastically reduces the flotation [96]. Cholesterol depletion in this cell line is responsible for misfolding of immature PrP, which may affect maturation of PrP^C^ and therefore PrP^Sc^ propagation. However, the mechanism of its involvement is only poorly understood.

Lovastatin treatment increased the overall half-life time of PrP^C^ which can increase PrP^Sc^ formation, because of the increased availability of PrP^C^ substrate. The initial step of *NH_2_*-terminal degradation of PrP^C^ which results in a *C*-terminal 17 kDa PrP fragment [98,99] (PrP C1) seems to occur within lipid rafts. Both the full-length PrP^C^ and PrP C1 displayed similar solubility in Triton X-100, suggesting that the membrane microenvironment for both these PrP^C^ species is the same. This finding also might imply that PrP^Sc^ formation increases, since the initial *N*-terminal degradation is protective against conversion, on the contrary, PrP^Sc^ is decreased [48]. This can be explained by the fact that lipid raft association is impaired, and for the conversion of PrP^C^ into PrP^Sc^ internalization to an intracellular compartment is required [36,37,41,42].

Furthermore, PrP^C^ association with lipid rafts is disrupted when cellular cholesterol levels are decreased using squalestatin [51], a selective inhibitor of squalene synthase known to catalyze the synthesis of squalene, a cholesterol precursor, or by using lovastatin [48,52]. PrP^Sc^ formation also was found to be decreased. This effect on PrP distribution in three different cell lines (ScN2a, SMB and GT1) was reversed by adding soluble cholesterol [51], suggesting that localization of different proteins, including PrP^C^, to particular cholesterol-enriched lipid rafts depends on the lipid constitution of these microdomains. However, lipid raft association of PrP^C^ is required for its transport to the cell surface. When this association was destroyed by cholesterol depletion in N2a cells using mevinolin, PrP^C^ was intracellularly retained in the Golgi compartment, and it was emphasized that given the importance of PrP^C^ cell surface localization for PrP^Sc^ propagation, this intracellular retention of PrP^Sc^ is responsible for the reduced PrP^Sc^ accumulation [52]. Despite impairing PrP^Sc^ formation, it appears that inhibition of cholesterol synthesis has other effects on neurons that depend on PrP^C^. Indeed, N2a cells treated with atorvastatin exhibited increased PrP^C^ levels in a time- and dose-dependent manner, which leads to an increase of neurite outgrowth [100]. This suggests that neurite outgrowth is mediated by PrP^C^ in a cholesterol-dependent manner.

#### 4.2.2. Cholesterol Esterification and Prion Formation

In addition to cholesterol synthesis and uptake, cholesterol esterification is of importance to control free cholesterol levels in the plasma membrane. Free cholesterol transferring to the ER is esterified by ACAT which enables cholesterol storage in cytoplasmic lipid droplets [101]. In this study, the chemical compounds TMP-153, FR179254 and YIC-C8-434 were used to inhibit ACAT activity, and toxicity was compared between prion-infected and non-infected cells. These drugs were more toxic to prion infected cells (ScGT1 and N2a cells), demonstrated by an increased level of active caspase-3. Adding cholesterol esters to cells did not reverse the ACAT-inhibitor mediated toxicity while adding free cholesterol increased it. This suggests that the ACAT-inhibitor mediated toxicity is related to an increase in free cholesterol content of cells instead of a decrease in cholesterol ester levels [101].

In another study, modulators of cholesterol ester (CE) formation such as Sandoz 58-035 and everolimus (EVE) were used to treat skin fibroblast derived from scrapie-infected sheep and 22L-N2a cells, respectively [102]. In skin fibroblasts from scrapie-infected sheep, CE accumulation was reduced by the different drugs. Treatment of 22L-N2a cells for 4 days resulted in a reduction of PrP^Sc^ accumulation, analysed by dot blot and illustrated by dose-response curves. It was also found that peripheral blood mononuclear cells and skin fibroblasts isolated from scrapie-infected sheep, infected mouse brains and infected N2a cells displayed an abnormal accumulation of cholesterol esters, suggesting that this can be used as a biomarker for prion infection [102,103].

#### 4.2.3. Cholesterol at the Plasma Membrane, PrP Isoforms and Prion Disease Progression

In contrast to complete cholesterol depletion achieved by treatment with statins and other inhibitors of cholesterol synthesis, several studies address the question how selective reduction of cholesterol at the plasma membrane affects PrP^Sc^ formation. Extracting membrane cholesterol from cells has been demonstrated to dissociate proteins from lipid rafts [104,105], and association of PrP with lipid rafts is necessary for the conversion of PrP^C^ to PrP^Sc^ [106]. To analyze the effect of membrane cholesterol extraction on prion propagation, N2a cells infected with the 22L strain were treated with β-cyclodextrin (β-CD), resulting in a reduction of PrP^Sc^ [107]. β-CD had already been shown to reduce the toxicity of Aβ peptide in AD [107] and to remove cholesterol from membranes. In addition, treatment of human microglial and mouse neuroblastoma cells with the polyene antibiotic filipin could inhibit the accumulation of PrP^Sc^ [24]. Indeed, filipin binds cholesterol at the membrane, destabilizes the lipid composition of the membrane and thus leads to the release of GPI-anchored-proteins. PrP-release from lipid rafts prevents endocytosis of PrP, reduces the amount of PrP substrate available for conversion, and thus, significantly decreases PrP^Sc^ accumulation [24]. In addition, Amphotericin B, another polyene antibiotic, was identified as therapeutic agent in scrapie already in the 1980s [108]. Amphotericin B interacts with cholesterol and thus impairs the lipid composition of membranes. Using prion-infected GT1-7 and N2a cells it was demonstrated that PrP^Sc^ formation is inhibited when cells are treated with Amphotericin B [109]. However, the effect of the drug is not maintained when treatment is omitted.

#### 4.2.4. Manipulation of Cholesterol in the Endocytic Pathway

Several studies using different techniques demonstrated that disruption of cholesterol balance leads to decreased levels of PrP^Sc^ formation [24,48,51,109,110,111]. Klingenstein and co-workers found that heterocyclic compounds such as quinacrine, which originally was used as an antimalarial drug, reduces PrP^Sc^ accumulation in prion-infected cells [112]. The authors suggested that the anti-prion effect of quinacrine was induced by cholesterol redistribution from the plasma membrane to late endosomes, leading to a destabilization of lipid rafts. To confirm their finding, they tested three known potent cholesterol-redistributing drugs U18666A, amiodarone and progesterone in ScN2a cells. Consistently, treatment of these cells with these compounds has resulted in reduction of PrP^Sc^.

In line with this, silencing expression of the NPC-1 gene, known to be mutated in Niemann-Pick type C disease and leading to an aberrant trafficking of cholesterol and late endosome/lysosome enlargement [113], using siRNA induces a significant reduction of PrP^Sc^ in ScN2a cells [111]. The same results were obtained when ScN2a cells were treated with U18666A, which is frequently used to induce a Niemann-Pick type C disease phenotype in cultured cells, characterized by accumulation of cholesterol in late endosomes/lysosomes [114]. Reduction of PrP^Sc^ levels upon U18666A treatment was confirmed by using two other cell lines, ScSN56 and ScGT-1. U18666A did neither inhibit primary infection and *de novo* formation of PrP^Sc^, nor impair the localization of PrP^C^ at the plasma membrane or its association with lipid rafts. It was shown that U18666A treatment results in an increase in PrP^Sc^ degradation [111], possibly by induction of autophagy, a cellular pathway which, upon drug-induced stimulation, leads to clearance of PrP^Sc^ [115,116,117]. PrP^Sc^ propagation could be rescued by overexpression of rab9, a small GTPase mediating vesicle shuttling between late endosomes and trans-Golgi network which alleviates cholesterol accumulation in U18666A treated cultured cells [118]. However, U18666A was not effective in mouse models of prion disease [119]. In line with this, recently tamoxifen (TAM) and its metabolite, 4-hydroxytamoxifen (OHT) were reported to increase lysosomal degradation of PrP^Sc^ in scrapie-infected GT1 and N2a cells [120]. This effect was ascribed to redistribution of PrP^C^, PrP^Sc^ and cholesterol into lysosomes.

In summary, induction of cholesterol accumulation in late endosomes/lysosomes results in a reduction of PrP^Sc^, likely due to an accelerated degradation rather than inhibition of *de novo* conversion.

#### 4.2.5. Cholesterol, a Potential Target for Treatment of Prion Diseases *in Vivo*

Given that inhibition of cholesterol synthesis has repeatedly been demonstrated to reduce PrP^Sc^ levels in prion-infected cells and that statins are commonly used to treat hypercholesterolemia in humans, several studies in rodent models of prion disease were initiated to explore the therapeutic potential of statins and other drugs that target cholesterol metabolism (summarized in Table 1).

**Table 1:** Effect of chemical compounds on cholesterol pathways and disease progression.

Compound	Model	Cholesterol level	Effect on PrP^C^/PrP^Sc^ or APP/Aβ level	References
Lovastatin	ScN2a cells	Depletion of cellular cholesterol	↓PrP^C^ degradation	[48]
	HaB cells		↓PrP^Sc^ accumulation	
Squalestatin	ScN2a cells	Reduction of cellular cholesterol	↓PrP^Sc^ accumulation	[51]
	SMB cells			
	ScGT1 cells			
Atorvastatin	N2a cells	Inhibition of cellular cholesterol	↑PrP^C^ level	[100]
Filipin	ScN2a cells	Sequestration of cholesterol and disruption of membrane structure	↓PrP^Sc^ accumulation	[24]
Amphotericin B	ScN2a cells	Interaction with cholesterol and disruption of membrane structure	↓PrP^Sc^ accumulation	[109]
	ScGT1-7 cells			
ACAT inhibitors	N2a cells	Inhibition of cholesterol ester formation (cholesterol relocation)	↓PrP^Sc^ accumulation	[102]
Simvastatin	C57BL/6 infected with ME7 (IC)	Cholesterol level unchanged	PrP^C^/PrP^Sc^ unchanged	[121]
			Increased survival time	
Simvastatin	C57BL/6 infected with ME7 (IC)	Cholesterol level unchanged	PrP^C^/PrP^Sc^ unchanged	[121]
			No significant effect	
Simvastatin	FVB/N infected with RML (IC/IP)	Cholesterol level unchanged	PrP^C^/PrP^Sc^ unchanged	[122]
			Increased onset of symptoms	
Simvastatin	C57BL/6 infected with 139A (IC)	Cholesterol level unchanged	PrP^C^/PrP^Sc^ unchanged	[123]
			Increased survival time	
Pravastatin	C57BL/6 mice infected with 139A (IC)	Cholesterol level unchanged	PrP^C^/PrP^Sc^ unchanged	[124]
			Increased survival time	
Amphotericin B	Syrian hamster infected with 263 K (IC/IP)	Interaction with cholesterol and disruption of membrane structure	↓PrP^Sc^ accumulation	[125,126]
			Increased survival time	
Amphotericin B	Scrapie infected mice and hamsters	Interaction with cholesterol and disruption of membrane structure	↓PrP^Sc^ accumulation	[127,128,129,130]
Simvastatin/Lovastatin	Rat Hippocampal neurons	Depletion of cellular cholesterol	↓Aβ production	[131]
	HEK 293 cells			
	SHSY5Y cells			
ACAT inhibitors	CHO cells hippocampal primary neurons	inhibition of cholesterol ester formation (cholesterol relocation)	↓Aβ production	[132]
ACAT inhibitors	transgenic APP-mice (London and Swedish mutation)	inhibition of cholesterol ester formation (cholesterol relocation)	↓Amyloid plaques	[133]
			↓Aβ production	
			Slightly improve of spatial learning	
Seladin 1	Rat hippocampal neurons	Decrease cholesterol level	↑Aβ production	[134]
	CHO cells			
Statins (cholesterol-lowering drugs)	The Rotterdam study (6992 Non AD subjects)	Cholesterol-lowering	Reduced risk of late-onset AD	[135]

Indeed, intracerebrally infected C57Bl/6 mice with the ME7 scrapie strain, at the same time orally treated with simvastatin demonstrated in a significantly longer incubation period and a delay in scrapie-induced loss of motor coordination. However, no significant difference in the survival times was seen in ME7-infected mice when simvastatin treatment was started at the onset of behavioral changes, suggesting that simvastatin treatment is only efficient when used in the preclinical phase of the disease in this model [121]. Both intracerebral and intraperitoneal administration of simvastatin to mice intracerebrally infected with RML resulted in a significant delay of disease progression and thus an increased survival time of these mice [122]. These effects seemed to be independent of decreased cholesterol levels and/or lipid properties of raft microdomains, and of the conversion of PrP^C^ into PrP^Sc^. Interestingly, the levels of PrP^Sc^ were even increased in simvastatin treated mice, which could be due to the longer life span of those mice, resulting in a longer prion replication period and/or because of the neuroprotective effect of simvastatin, which keeps neurons alive that are able to accumulate more PrP^Sc^. Possibly, reduced levels of cholesterol as expected upon treatment are counter-balanced by an increase of cellular cholesterol levels induced by prion infection (see below, Section 4.3). Thus, the simvastatin effect might be only due to the neuroprotective effects of the compound rather than changes in cholesterol metabolism [136]. In line with this is another study, where treatment of 139A-infected mice with simvastatin was started at 100 days post-infection. Prion-infected and treated mice exhibited a significant delay in prion disease progression and an extended time of survival [123]. Again, the level of PrP^Sc^ was not significantly affected. In another study, 139A scrapie-infected mice were orally treated with pravastatin, which is, in contrast to simvastatin, a hydrophilic HMGCR inhibitor that can be found in measurable amounts in the CNS upon oral application. Treated mice exhibited a delay in disease symptoms as well as prolonged survival times. PrP^Sc^ accumulation and glycotype pattern as well as astrocytosis in the brains of those mice were unchanged [124]. Overall, it appears that the effects of statins *in vivo* are not due to a reduction of cholesterol or an inhibition of PrP^Sc^ propagation, but their neuroprotective effects may lead to the observed delay in disease onset.

Furthermore, Syrian hamsters infected with 263K scrapie strain and treated with Amphotericin B exhibited delays in the incubation period [125,126,137]. In intracerebrally prion infected mice [129,130] or Syrian hamsters [131], treatment with Amphotericin B or MS-8209 prolonged incubation time of disease and reduced PrP^Sc^ formation and glial fibrillary acidic protein (GFAP) expression, even when started at later stages post inoculation, when PrP^Sc^ accumulation in the brain is significant. MS-8209 is a less-toxic derivative of Amphotericin B which also has an antiprion effect. *In vitro*, in cerebellar granule neurons- (CGN-) infected model, treatment early after prion exposure (3 dpi) with MS-8209 was shown to be efficient against PrP^Sc^ accumulation of a wide range of prion strains from different species, even though the efficacy was variable and strain-dependent [138]. Late treatment (11 dpi) with MS-8209 resulted only in a moderate inhibitory effect of PrP^Sc^ accumulation in CGN-cultures, infected with the ovine prion strain 127S. In addition, MS-8209 treatment results in longer life spans of transgenic tg52NSE mice which have a neuron-restricted hamster PrP expression when infected with 263K scrapie strain but not with Drowsy strain [127]. MS-8209 treatment was more efficient in prolonging the incubation period of C506M3 scrapie strain in mice than that of a BSE-adapted agent in C57BL/6 mice. Interestingly, hamsters infected with the 263K scrapie strain, treated with MS-8209, showed a much longer incubation time. Furthermore, PrP^Sc^ accumulation was delayed in the brains of scrapie-infected mice [130] but not significantly reduced in BSE-infected mice [128]. However, the direct or indirect mechanisms of these drugs remain unclear. Although in some cases promising results were achieved, the effects are critically dependent on the prion strain used for infection.

### 4.3. How Does Prion Infection Interfere with Cholesterol Metabolism?

While many studies characterized the involvement of cholesterol in prion propagation, more recent studies pointed out that there is an inextricable relationship between prion infection and cholesterol metabolism. Using microarray analysis on neuronal cells infected with 22 L prions an up- regulation of genes involved in the cholesterol pathway was demonstrated. Sterol regulatory element-binding protein (SREBP2), a transcription factor closely involved in the transcription of genes associated with cholesterol biosynthesis and cholesterol up-take, was found to be activated during prion infection. This resulted in increased levels of both total and free cholesterol, suggesting a link between prion propagation and cholesterol balance [139]. This response to prion infection appears to be specific for neurons [139] and is also apparent in prion-infected mice at a preclinical stage of the disease [140]. When the effects of prion infection on cholesterogenic gene expression of primary neurons, astrocytes and a microglial cell line were analyzed, only neurons responded with an up-regulation. In astrocytes, no changes in cholesterogenic gene expression were found, whereas in a microglial cell line prion infection resulted in a down-regulation of cholesterol synthesis [139]. Of note, the membrane fluidity in prion-infected cultured cells was shown to be reduced [141], and it was demonstrated that prion-infected cells sequester cholesterol in cellular membranes [142]. These findings may be linked to the described up-regulation of cholesterol synthesis.

Surprisingly, ABCA1 was found to be increased during prion infection in both cell culture and mouse models of prion infection [143,144,145,146]. ABCA1 is a membrane-associated protein involved in cellular cholesterol export. Its localization under normal conditions is known to be mainly at the cell membrane. Upon prion infection, ABCA1 was overexpressed even though the level of free cholesterol in infected cells was increased. However, ABCA1 was found internalized during prion infection. The latter explained the increased level of free cholesterol in infected cells. This effect was reversed when prion infection was cured. When ABCA1 was overexpressed, PrP^Sc^ formation was reduced, probably through the cholesterol pathway since the levels of PrP gene expression or protein amounts were unchanged [146]. One possible explanation for the ABCA1 enhancement is the cross-talk between cholesterol and the abnormal prion protein. During prion infection cholesterol synthesis is increased, and this imbalance in cholesterol homeostasis could lead to an overexpression of different genes involved in the cholesterol pathway in order to regulate this impairment. These results clearly demonstrate that there is a mutual relationship between cholesterol balance and prion infection. However, it is unclear whether an increase in cholesterol levels is necessary to enable persistent prion propagation.

Since prion diseases are still incurable neurodegenerative disorders, manipulating the cholesterol pathway during prion infection could be of interest in designing new therapeutic approaches.

## 5. Cholesterol in Other Neurodegenerative Disorders

The importance of cholesterol balance in the brain is revealed by severity and complexity of pathologies linked to the disruption of cholesterol homeostasis. Cholesterol accumulation is involved in several rare neurodegenerative diseases, for example Cerebrotendinous Xanthomatosis [147,148], Smith-Lemli-Opitz syndrome [149,150] and Niemann-Pick type C disease [151]. Beyond these rare pathologies, it is also involved in the more common neurodegenerative diseases which are associated with accumulation of protein aggregates, such as Alzheimer’s disease (AD), Huntington’s disease (HD) and Parkinson’s disease (PD). In neurodegenerative diseases, cholesterol and AD relationship is the most described in the literature, this is why we will focus our discussion on AD in the following session.

### 5.1. Cholesterol and AD

AD is a neurodegenerative disorder characterized by progressive and irreversible memory impairments, associated with cognitive decline. The pathological hallmarks of AD are extracellular amyloid plaques of amyloid β (Aβ) peptide and intracellular neurofibrillary tangles. Aβ peptide is produced by proteolytic cleavage of the Alzheimer precursor protein (APP; [152]), a type I transmembrane protein that can be subjected to different cleavages processed by three proteolytic enzymes: α-, β- and γ-secretase. APPα-secretase cleavage occurs at the plasma membrane and it represents the first step of the non-amyloidogenic pathway [153], as it cleaves within the Aβ peptide. An alternative pathway for APP processing is the amyloidogenic pathway which includes processing by β- and γ- secretase [154] and leads to Aβ peptide production.

In fact, APP associated with lipid rafts is internalized into endosomes where APP is cleaved by the β-secretase BACE1. This cleavage generates an extracellular soluble fragment and a cell membrane bound fragment known as C99. The latter will be cleaved by a γ-secretase and this releases the APP intracellular domain (AICD) and the amyloid peptides Aβ-40 and Aβ-42.

Early onset AD is associated mainly with mutations in the APP gene favoring the amyloidogenic pathway. However, other genetic (ApoE4 allele) or environmental factors (gender, high blood pressure, and hypercholesterolemia) can promote onset of the disease.

### 5.2. Cholesterol in the Brains of AD Patients

Emerging evidence has highlighted that cholesterol balance plays a role in the pathogenesis of AD and that high cholesterol levels may be a risk factor for the development and the progression of the disease, although the underlying molecular mechanisms are still not clear.

Cutler and colleagues [155] demonstrated that cholesterol is increased in the frontal gyrus but not in the cerebellum (this tissue is not affected during AD) of AD patients. Cholesterol is enriched in the plasma membranes isolated from brains of AD patients at different stages of AD. Brains from moderate decline-patients (stage 4) exhibited increased cholesterol levels in comparison with controls, and brains from severe decline patients (stage 6) exhibited significantly increased cholesterol levels when compared to brains from mild decline patients (stage 3). Thus, cholesterol levels increase throughout the course of clinical disease. This was confirmed by Xiong and co-workers [156]. Cholesterol might be enriched around senile plaques, the extracellular deposits of beta amyloid, and abnormal neurites. The authors thus suggested that an increase in cholesterol retention in the CNS is responsible for an increase in Aβ peptide formation due to an increased processing by β- and γ-secretases ([153] and discussed below). Furthermore, it was reported that there is a significant correlation between Aβ peptide formation and lipid composition of lipid rafts, suggesting that variations in the lipid composition are of importance in AD pathology [157,158,159]. Docosahexaenoic acid (DHA), known to promote the non-amyloidogenic pathway and to influence cholesterol homeostasis [160,161], is decreased in lipid rafts in neuronal membranes in different brain areas [162] and also in AD brains compared to control [163].

Marquer and colleagues [164] found that high cholesterol levels at the plasma membrane in primary cultured neurons and HEK cells lead to an increased Aβ production. This effect on Aβ production is due to an improved accessibility of APP for BACE1 cleavage. They elegantly demonstrated that cholesterol loading does not affect BACE1 activity directly [156], but rather induces APP redistribution and clustering in lipid rafts at the plasma membrane where BACE1 is present. This gathering of BACE1 and its substrate is rapidly followed by internalization to endosomes where APP cleavage will occur, resulting in an increase in Aβ production [164]. This result confirmed an earlier study where it was shown that targeting BACE1 inhibitors to lipid rafts leads to a decrease in hippocampal Aβ levels in an AD mouse model [165].

Increased BACE1-mediated APP cleavage also leads to increased release of AICD, which down-regulates low density lipoprotein-related protein 1 (LRP-1) transcription and decreases the amount of LRP1 receptor which is responsible for exogenous cholesterol capture at the plasma membrane [166]. Moreover, Aβ-40 inhibits HMGCR, the rate-limiting enzyme in cholesterol biosynthesis [167]. Furthermore, it was shown that a severe decrease in Aβ peptide in fibroblasts of transgenic presenilin knock-out mice causes an increase of cellular cholesterol levels [167]. These results suggest that an increase in cholesterol at the plasma membrane induces an increase of APP endocytosis which leads to formation of Aβ peptide and AICD, which in turn decreases levels of intracellular cholesterol. Thus, the liaison between APP and cholesterol ultimately results in a decrease of cellular cholesterol levels.

### 5.3. Cholesterol Reducing Agents in AD Models

The findings described above suggest that lowering cellular cholesterol levels will counteract Aβ peptide formation, and several modulators of cholesterol were tested.

Methyl-β-cyclodextrine (MβCD) is known to decrease cholesterol in the plasma membrane. *In vitro* when using MβCD in cultured primary neurons, this treatment decreases Aβ peptide formation [153,168,169], suggesting that this process is influenced by lipid composition in lipid rafts [170]. Several mechanisms were proposed to explain this decrease, e.g., an increase of α-secretase activity or that an impairment of lipid rafts leads to reduced APP cleavage and thus to a diminished Aβ peptide formation [171]. In contrast, another study revealed an increase in Aβ peptide formation when 20% of cholesterol is depleted at the plasma membrane [134]. The effects of statin treatment on development of AD is still controversially discussed. Whereas several studies have revealed that patients treated with statins exhibited an about 70% decreased prevalence of AD [172,173,174], others did not confirm this finding [175,176,177]. It also demonstrated that the decreased risk for AD was not associated with the capacity of statins to cross the BBB. Thus, statins seem to act in the periphery rather than in the CNS. Beneficial effects of statins are also evident from studies in transgenic mice. Indeed, in APP-transgenic mice, statins were shown to decrease Aβ peptide formation [178,179,180]. In contrast, a diet high in cholesterol increases the number of senile plaques [181]. However, because cholesterol levels were not found to be affected by statins in rodents [182], further evidence is required to determine the role of statins in a cholesterol-dependent amyloidogenic pathway.

In addition to their function in decreasing cholesterol level, statins have pleiotropic neuroprotective effects [183] and side-effects such as increase of HDL, decrease of triglycerides, and impairment of plasma apolipoprotein concentration [184].

Using a neuronal cell line overexpressing APP, statins were found to increase α-secretase activity and thereby decrease Aβ peptide secretion [131,185] and the inflammatory response [186]. Statins, as well as MβCD, decrease cholesterol at the outer leaflet of the plasma membrane, leading to a decreased Aβ peptide formation [187]. The authors suggested a re-organization of cholesterol-enriched lipid rafts that decreases processing of APP by BACE1, a hypothesis that was confirmed later [164]. Another explanation is that statins interfere with isoprenylation, leading to APP accumulation in the proteasome, thereby decreasing amyloid peptide formation [188].

It is not necessary to really change cholesterol concentrations to impair Aβ peptide formation. Indeed, a re-localization of cholesterol in the cell is sufficient to disrupt APP cleavage [164]. Inhibition of ACAT reduced amyloid pathology and improved cognitive performance in mice [133]. Consequently, several pharmacologic inhibitors were developed and tested in AD [189]. ACAT-inhibitors CP-113,818 and CI-1011 were shown to reduce Aβ generation *in vitro* and *in vivo* [132,133] by affecting APP-α-, β- and γ-cleavage [166]. Nevertheless, BACE1 or γ-secretase activities *in vitro* are not altered by CP-113,818 [133]. It appears likely that reduced APP processing, upon inhibition of ACAT, is mediated by an indirect modulation of secretase activities, probably by disturbing APP trafficking. In fact, CP-113,818 treatment, using cell models *in vitro* as well as using hAPP transgenic mice *in vivo*, has been demonstrated to delay APP maturation in the early secretory pathway by retaining immature APP in the ER. Newly synthesized APP is thus unavailable for cleavage and this leads to reduced Aβ peptide production [190].

### 5.4. Genes that Affect Cholesterol Metabolism and AD

Cholesterol 24-hydroxylase, encoded by the CYP46A1 gene, converts cholesterol to 24S-hydroxycholesterol and allows its transport from the brain to the liver where it will be eliminated. Overexpression of this protein decreases the amount of Aβ peptide and senile plaques, and improves cognitive performance in the mouse model. The mechanism is still poorly understood, but it seems to occur through a decrease of γ-secretase activity. In line with this, in mouse models, cholesterol 24-hydroxylase inhibition increases the formation of Aβ peptides in the brain. A direct link to cholesterol levels in the brain can potentially explain these results [191]. Recently, it was found that crossing cholesterol 24-hydroxylase knock-out mice with a mouse model of AD does not affect amyloid formation. Overall, the cerebral cholesterol levels were not different between these mice and used controls. Interestingly, the excretion level of cholesterol from the brain was decreased by 50%, counterbalanced by a 50% decrease of *de novo* cholesterol synthesis. Thus, the authors conclude that a reduced *de novo* synthesis of cholesterol in the brain does not alter AD progression even though it expands the lifespan of mice [192].

SELective AD INdicator-1 gene (seladin-1) was identified by mRNA comparisons between affected (cortex, hippocampus) and non-affected brain regions from AD patients [193]. Seladin expression was found significantly down-regulated in affected brain tissues. The function of seladin is to convert desmosterol into cholesterol via its 3-β-hydroxysterol ∆-24-reductase activity. Overexpression of seladin increases cerebral cholesterol in mice [194], and in parallel, increases resistance to toxic effects of Aβ peptide in human neuroblastoma cells [195]. In transgenic seladin-1 deficient mice, APP processing by β-secretase and thus the amyloidogenic pathway is enhanced. This can be the result of a decrease in cholesterol [194]. In the same study, overexpression of seladin-1 in neuroblastoma cells was found to increase cholesterol levels and to reduce APP processing. Along the same line, moderate reduction of membrane cholesterol found in AD patients, paradoxically, seems to facilitate the interaction between APP and BACE1-β-secretase and consequently increasing β-amyloid peptide production [134]. However, the mechanism underlying this cholesterol-loading effect on APP processing is still not clear. In fact, increased Aβ production would result from either increased BACE-1 activity or increased APP access to BACE-1 in the plasma membrane or in the endocytic compartment. The use of cell models overexpressing APP in some previous studies [131,169,171] may explain the contradictory results shown in these studies.

These and other results demonstrate that cholesterol is important for inducing Aβ peptide toxicity and for its binding to the plasma membrane [196]. Thus, seladin-1 down regulation may affect membrane permeability and increase cell vulnerability to Aβ peptide. The neuroprotective role of seladin-1 clearly emphasizes the importance of an optimal amount of cholesterol in cells for brain homeostasis. However, these results which demonstrate a protective effect of excess cholesterol are also related to several other known functions of seladin [197], such as inhibition of apoptosis, neurogenesis, and growth factor regulation.

ApoE is known to be the strongest risk factor for the development of sporadic late-onset AD. Indeed, the incidence of AD is significantly increased in individuals with the ε4 allele [198,199]. Lipoproteins that contain ApoE are produced by glial cells and deliver cholesterol and essential lipids to neurons through their interaction with the LDL-R. Mechanisms by which ApoE is thought to be a risk factor in AD are still poorly known, but several pathways have recently been identified *in vivo* and *in vitro*. ApoE4 appears to be a less effective cholesterol transporter than ApoE3 [200] and therefore, less efficient in promoting cholesterol efflux from neurons and astrocytes [201]. Human ApoE4 knock-in mice exhibited an increase in cholesterol at the outer leaflet of the plasma membrane [202] in comparison to knock-in ApoE3 mice. ApoE was found to interact with Aβ [203]. Tiraboschi and co-workers [204] demonstrated that the number of ε4 alleles in AD patients determines neuritic plaque accumulation. More recently, it was found that ApoE4 increases the level of fibrillar Aβ accumulation in cognitively normal older people in a dose-dependent manner [205], as well as in AD patients [206]. Therefore, it seems that ApoE4 accelerates the appearance of amyloid deposits and promotes its fibrillar aggregation.

LRP1, known to be a receptor of ApoE, can also interact with APP and modulate its trafficking by increasing APP endocytosis and thus increase Aβ-production [207]. In addition, statin treatment increases LRP1-mediated clearance of Aβ [208]. Interestingly, it was reported that the beneficial effect of statin treatment in AD is associated with subjects harboring the ε4 allele [176]. However, the mechanism by which ApoE modulates cholesterol balance in AD is still unknown.

## 6. Conclusions

It is striking that in both prion infection and AD, cholesterol plays a critical role in the formation of the associated protein aggregates. In addition, prion infection and Aβ peptide accumulation interfere in neuronal cholesterol metabolism, and it appears that in both conditions cellular cholesterol levels are increased, which can be toxic for cells. Therefore, questions arise whether cholesterol imbalance is involved in neurodegeneration, or how this dysregulation can impact the function of neurons. Cholesterol is an important regulator of membrane fluidity and function. It is involved in neurotransmission processes, and it promotes synaptogenesis, in which it could be a limiting factor [209]. Cholesterol is critical for the formation of synaptic vesicles containing neurotransmitters; it forms lipid rafts which are important on the presynaptic membrane for exocytosis of neurotransmitter, and on the post synaptic membrane, where the receptors of neurotransmitters are embedded [210]. With all these functions, it is obvious that cholesterol is an important regulator of neuronal activity and any dysregulation in its metabolism might have serious consequences on brain function. Therefore, it is reasonable to argue that an imbalance in cholesterol homeostasis may be one factor accountable for synaptic dysfunction and neurodegeneration in prion diseases and AD. Therefore, given that also pharmacological challenges such as the necessity for a drug to be BBB permeable are identical in prion diseases and AD, it may be possible that drugs targeting the cholesterol pathway are identified that are beneficial for the treatment of both diseases.
